# Allosteric Probe-Based Colorimetric Assay for Direct Identification and Sensitive Analysis of Methicillin Resistance of *Staphylococcus aureus*

**DOI:** 10.4014/jmb.2312.12042

**Published:** 2024-02-19

**Authors:** Juan Chu, Xiaoqin Zhao

**Affiliations:** Department of Dermatology, Zhuji Affiliated Hospital of Shaoxing University, Zhuji City, Zhejiang Province 31180, P.R. China

**Keywords:** Methicillin-resistant *Staphylococcus aureus* (MRSA), G-quadruplex, colorimetric assay, skin and soft tissue infections (SSTIs)

## Abstract

The accurate and rapid detection of methicillin-resistance of *Staphylococcus aureus* (SA) holds significant clinical importance. However, the methicillin-resistance detection strategies commonly require complicated cell lysis and gene extraction. Herein, we devised a novel colorimetric approach for the sensitive and accurate identification of methicillin-resistance of SA by combining allosteric probe-based target recognition with self-primer elongation-based target recycling. The PBP2a aptamer in the allosteric probe successfully identified the target MRSA, leading to the initiation of self-primer elongation based-cascade signal amplification. The peroxidase-like hemin/G-quadruplex undergo an isothermal autonomous process that effectively catalyzes the oxidation of ABTS^2-^ and produces a distinct blue color, enabling the visual identification of MRSA at low concentrations. The method offers a shorter duration for bacteria cultivation compared to traditional susceptibility testing methods, as well as simplified manual procedures for gene analysis. The overall amplification time for this test is 60 min, and it has a detection limit of 3 CFU/ml. In addition, the approach has exceptional selectivity and reproducibility, demonstrating commendable performance when tested with real samples. Due to its advantages, this colorimetric assay exhibits considerable potential for integration into a sensor kit, thereby offering a viable and convenient alternative for the prompt and on-site detection of MRSA in patients with skin and soft tissue infections.

## Introduction

Skin and soft tissue infections (SSTIs) are common in postoperative care, accounting for a large percentage of infections requiring hospitalization, and are associated with substantial morbidity. Prevention and early-diagnosis of SSTIs is crucial for the postoperative care of a variety of diseases, especially for these immunocompromised patients. SSTIs can be caused by diverse microorganisms, most commonly bacteria. Among them, *Staphylococcus aureus* (*S. aureus*) is the most common bacteria causing purulent skin and soft tissue infections. The methicillin-resistance of *S. aureus* complicated the nursing of patients with SSTIs. Methicillin-resistant *Staphylococcus aureus* (MRSA) infections have historically been linked to those with impaired immune systems in healthcare settings [1, 2]. The expeditious identification of MRSA directly from clinical samples holds significant significance in acquiring data regarding the susceptibility of antimicrobial drugs. The *mecA* gene, which is located in staphylococcal cassette micro chromosome, is responsible for conferring methicillin resistance in bacteria through coding penicillin binding proteins 2a (PBP2a) protein, which serves as a substitute for other penicillin binding proteins in the process of crosslinking peptidoglycan chains. Therefore, the detection of the *mecA* gene or its corresponding protein PBP2a is employed as a supplementary diagnostic method to promptly confirm the presence of methicillin resistance.

The conventional techniques employed for MRSA testing involve colony count and combining drug susceptibility test [3, 4]. These technologies generally require a duration of 2 to 4 days for the outcomes. Therefore, various culture-independent techniques have been devised to expedite the identification of MRSA, such as enzyme-linked immunosorbent assay (ELISA) and polymerase chain reaction (PCR) [5-7]. Nevertheless, the application of these methods targeting *mecA* gene detection is still restricted by high cost, low accuracy, time-consuming, complicated sample pre-treatment (complicated gene extraction procedures).

Aptamers, also called chemical antibodies, are single-stranded DNA or RNA that can form particular three-dimensional structures to bind to different molecules with high specificity and affinity [8]. The identification of the PBP2a aptamer has facilitated the direct detection of MRSA [9]. In contrast to conventional antibodies, aptamers exhibit enhanced stability and offer more adaptability in preparation and modifications. Aptamer-based MRSA sensing systems have been developed by combining with different signal transducers, including fluorescence, colorimetry and electrochemistry [10-12]. Among them, the colorimetric method has attracted abundant attention because it offers a simply visualized way to detect analytes by the naked eye [13]. Using AuNPs as an indicator, an aptamer-AuNPs based method for duplex identification of target was established and can well identify its target from interfering bacteria [14]. Despite the AuNP-based sensors being rapid and portable, they still suffer from low sensitivity and poor stability in real sample detection. By integrating with isothermal amplification strategies, the detection sensitivity of the approaches was significantly improved. For example, Zhen Li *et al*. proposed a dual-functional aptamer based method for sensitive MRSA detection based on CRISPR-Cas12a assisted rolling circle amplification (RCA) [15]. However, the RCA reaction is fixed on the magnetic bead surface and lacks enough amplification efficiency.

In this work, an allosteric probe based-target recognition and self-primer elongation initiated cascade signal amplification strategy is devised to develop a rapid and simple colorimetric method for methicillin resistance analysis of *S. aureus*. The self-primer elongation initiated cascade signal amplification can generate enough peroxidase-like hemin/G-quadruplex to produce a highly sensitive colorimetric readout signal in the presence of MRSA. The proposed method displayed a low limit of detection and a high selectivity to target bacteria by utilizing PBP2a aptamer based accurate MRSA identification and high-efficient signal amplification. This approach has significant potential for on-site detection of MRSA in SSTIs, and it can also be easily adapted to the detection of clinical samples.

## Experimental Section

### Reagents and Materials

All the oligonucleotides used were synthesized by Shanghai Sangon Biological Engineering Technology & Services Co. Ltd. (China) and purified by using a high-performance liquid chromatography (HPLC) system. The details of oligonucleotide probes used in this work are listed in Table 1. The two probes, including the H1 probe and the H2 probe were designed with hairpin structure. The enzymes for the self-primer elongation process, including the phi29 DNA polymerase, endonuclease, and the deoxyribonucleotide mixture (dNTPs) were obtained from Thermo Scientific Co. Ltd. (China). The reagents for the color reaction, including the 2,2’-azinobis(3-ethylbenzothiozoline)-6-sulfonic acid (ABT^S^) were purchased from Sigma-Aldrich (China). Briefly, characterized bacteria strains were purchased from American Type Tissue Culture Collection (ATCC) while clinical isolated were kindly provided by microbiology research and hospital labs, including the MSSA (methicillin sensitive *Staphylococcus aureus*, CCTCC AB91118), *Escherichia. coli* (*E. coli*, ATCC 43896), *S. pneumoniae* (ATCC 50761), and *P. aeruginosa* (ATCC 19115). The suspected MRSA colonies were isolated from clinical samples at a hospital in Shaoxing. The MICs of the MRSA strains were determined by the E-test method. In details, the MRSA strains were divided into three groups according to the MICs (The definition of low-medium-high level groups were high: more than 4 μg/ ml; middle: 1.5~3 μg/ ml; low: lower than 1 μg/ ml).

### Self-Primer Elongation Based Cascade Signal Amplification

For MRSA detection, 1 U/L of phi29 DNA polymerase (2 μl), 2 U/L of Nt.BbvCI (2 μl), 200 μM of dNTPs (2 μl), 100 nM of the related probes (H1 and H2 probe) (5 μl), and various concentrations of MRSA (5 μl)were added to a tube containing 29 μl of PBS buffer solution. The mixture was incubated at room temperature for 60 min.

### Color Reaction

For the color reaction, 50 μl of the self-primer elongation based cascade signal amplification product was diluted with a 4-(2-hydroxyethyl)-1-piperazineethanesulfonic acid (HEPES) buffer (10 mM HEPES, pH 7.4) to 200 μl. Under the optimal conditions, the final concentrations of hemin, ABTS and H_2_O_2_ in the solution were 1 μM, 2 mM and 0.5 mM, respectively. After 5 min oxidation by H_2_O_2_, the color induced by the oxidation of ABTS^2−^ to ABTS•− could be observed, and UV–vis absorption spectra were recorded from 390 nm to 510 nm (with a peak value at 415 nm). The peak value at 415 nm of each sample collected using UV–vis spectrometer was recorded as the absorption intensity.

## Results and Discussion

### The Working Mechanism of the Proposed Approach for MRSA Detection

**Fig. 1:**
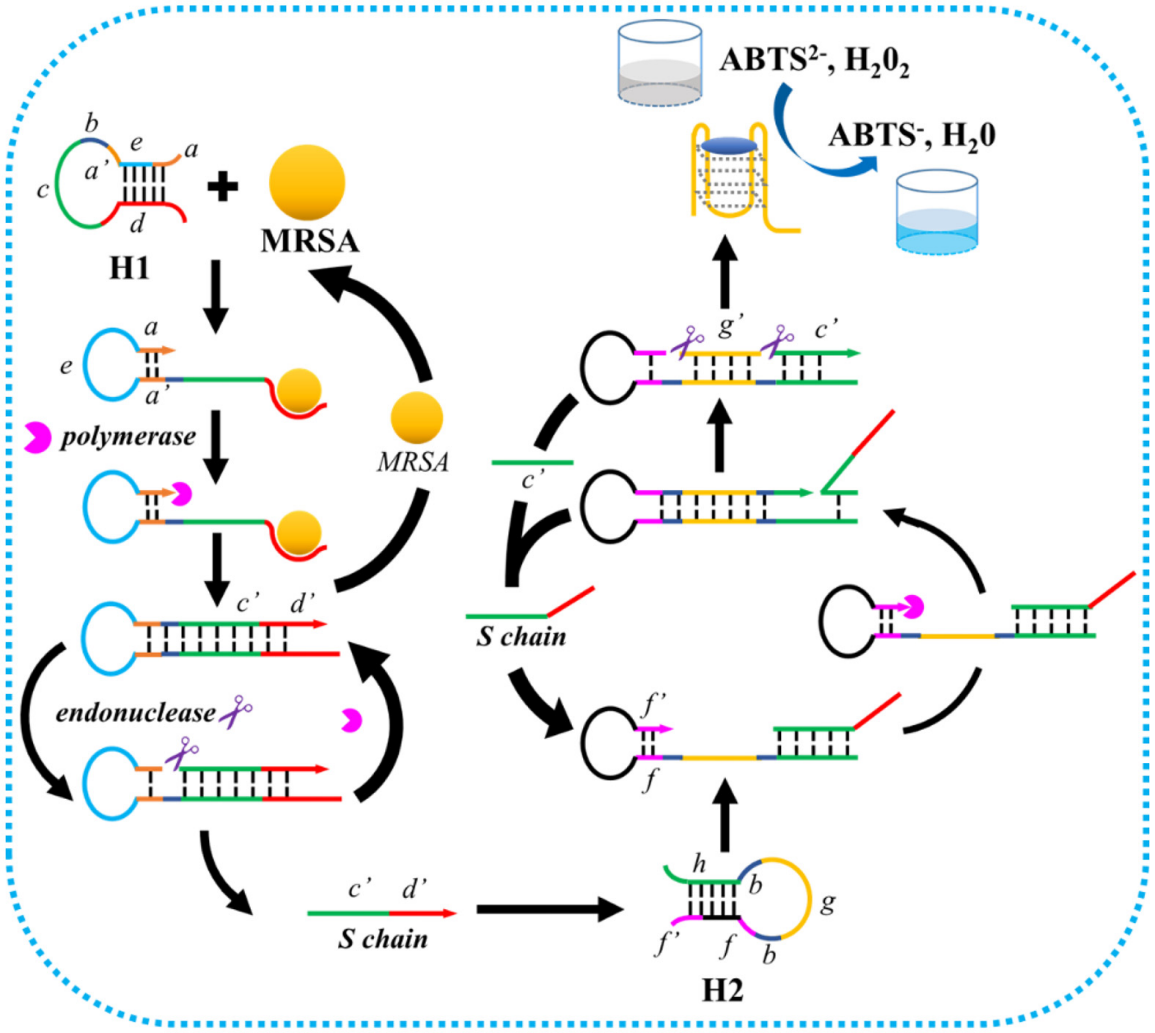
The working mechanism of the proposed approach for MRSA detection. H1 probe identifies target bacteria and forms a self-primer structure (“a” and “a’” fragment). With the “a” fragment as the primer, chain extensions and displacements are initiated to produce numerous S chain. The S chain induces H2 probe based signal amplification and color reaction.

The working principle of the proposed colorimetric approach is shown in Fig. 1. In this method, an allosteric probe (H1) is designed with hairpin structure and contains six functional sections, including the “a”, “b”, “a’”, “c”, “d”, and “e”. In detail, the “a” section is complementary with the “a’” section, “b” section is capable of transcribing endonuclease recognizing sites, “c” section transcribes “c’” which initiates subsequent signal amplification, “e” section is partially complementary with the “d” section, and the “d” section is the PBP2a aptamer. When MRSA exists in the sensing system, “d” section in the H1 probe binds with the PBP2a protein on the surface of MRSA and disassociates with the “e” section. Afterwards, the “a” hybridizes with the “a’” section and works as a primer to initiate chain extension under the assistance of DNA polymerase, transcribing the “b’”, “c’”, and “d’” sections. The endonuclease recognizes and cleaves the “b’” section, forming a nicking site. The DNA polymerase extents the “a” section and released S chain (containing “c” and “d”). The S chain binds with the “h” section and unfolds the H2 probe. The released “f ” section binds with the “f ’” section and initiates a next signal amplification process. With the “f” as primer, two endonuclease recognizing sites, “c’”, and “g’” sections were transcribed. Under the cooperation of DNA polymerase and endonuclease, large amount of “c’” and “g’” sections were released to the system. The “c’” section unfolds a next H2 probe to form cascade signal cycles, and the “g’” section which can fold into G-quadruplexe, catalyzing the oxidation of 2,2’-azino-bis (3-ethylbenzthiazoline-6-sulfonic acid) (ABTS), producing a blue-green colorimetric output signal, which could be monitored simply by the naked eye or with a spectrophotometer. With this strategy, the rapid and sensitive detection of MRSA with great selectivity was achieved.

### Feasibility Analysis of the Established Approach

**Fig. 2:**
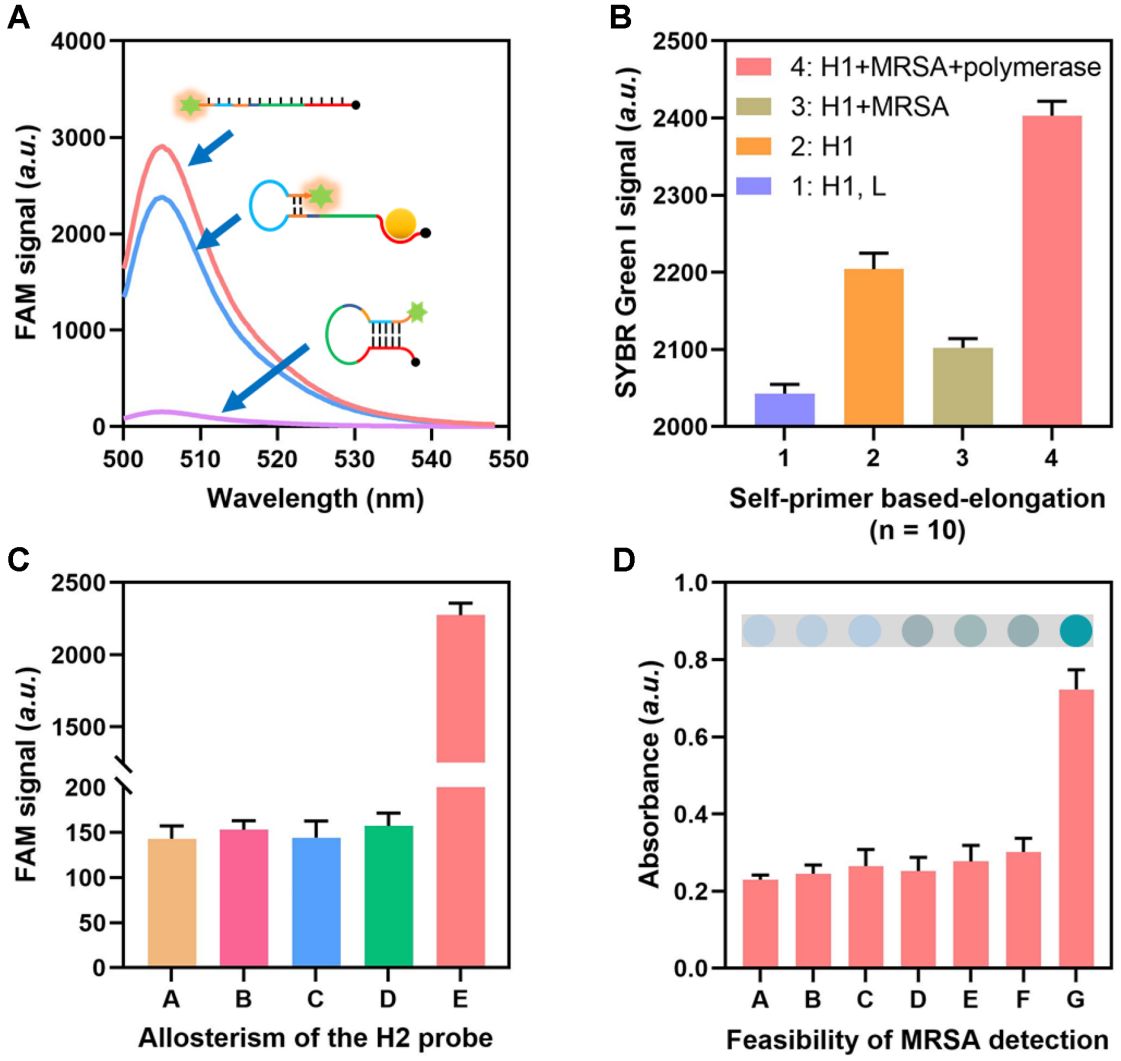
Feasibility of the proposed approach. (**A**) Fluorescence intensities of the FAM labeled H1 probe before and after assembly to hairpin structure. (**B**) SYBR Green I signals of the H1 probe during the self-primer elongation process. (**C**) FAM signals of the H2 probe during the self-primer elongation based signal recycling. “A” group, H2 probe; “B” group, H2 probe+ MRSA; “C” group, H2 probe+ MRSA+ DNA polymerase; “D” group, H2 probe+ MRSA+ DNA polymerase+ H1 probe; “E” group, H2 probe+ MRSA+ DNA polymerase+ H1 probe+ endonuclease. (**D**) Absorbance of the approach when essential components existed or not. “A” group, Blank control; “B” group, without MRSA; “C” group, without H1 probe; “D” group, without H2 probe; “E” group, without DNA polymerase; “F” group, without endonuclease; “G” group, with all.

The H1 probe was constructed into a hairpin conformation via annealing, a process that is important for MRSA identification and the initiation of subsequent cascade signal cycles. In this study, we initially evaluated the assembly of the H1 probe using a fluorescence assay. In detail, the H1 probe was labeled with FAM and BHQ (a quenching moiety) at its two terminus. In the linear state (H1, L), the FAM signal exhibited high intensity, which was subsequently suppressed by the BHQ when the linear H1 probe was configured into a hairpin structure. In the present of MRSA, the H1 probe’s “d” section forms a binding interaction with MRSA, resulting in the dissociation of FAM from BHQ. Consequently, the FAM signal reappeared, as depicted in Fig. 2A. The specificity of the H1 probe to PBP2a is tested through applying it to detect PBP2a, PBP1, and PBP3. The results in Fig. S1 showed a significantly elevated fluorescence signal of the H1 probe only when PBP2a existed. On the contrary, no significant fluorescence signals were observed when the H1 probes were incubated with PBP1 and PBP3, suggesting the high selectivity of the H1 probe to PBP2a. The utilization of SYBR Green I, a fluorescent dye known for its high sensitivity and precise recognition of double-stranded DNA (dsDNA), is employed in the characterization of DNA polymerases’ role in facilitating strand elongation. The findings depicted in Fig. 2B demonstrate that the SYBR signal was only heightened when both DNA polymerase and MRSA were present in the system. This suggests that the presence of both the target bacteria and DNA polymerase is necessary for the elongation of the DNA strand. The assembly of the H2 probe was further assessed using a fluorescent test, as depicted in Fig. S2. The findings depicted in Fig. 2C demonstrate the viability of the self-priming mechanism of the H2 probe. In the presence of MRSA, DNA polymerase, and endonuclease, a significantly heightened level of fluorescent signals was observed, suggesting the production of the S chain and the unfolding of the H2 probe. A colorimetric assay was conducted to assess the viability of the whole method. The observed alterations in color demonstrated that the presence of MRSA, H1 probe, H2 probe, DNA polymerase, and endonuclease were necessary components within the sensing system (Fig. 2D).

### Optimizing the Experimental Parameters

**Fig. 3:**
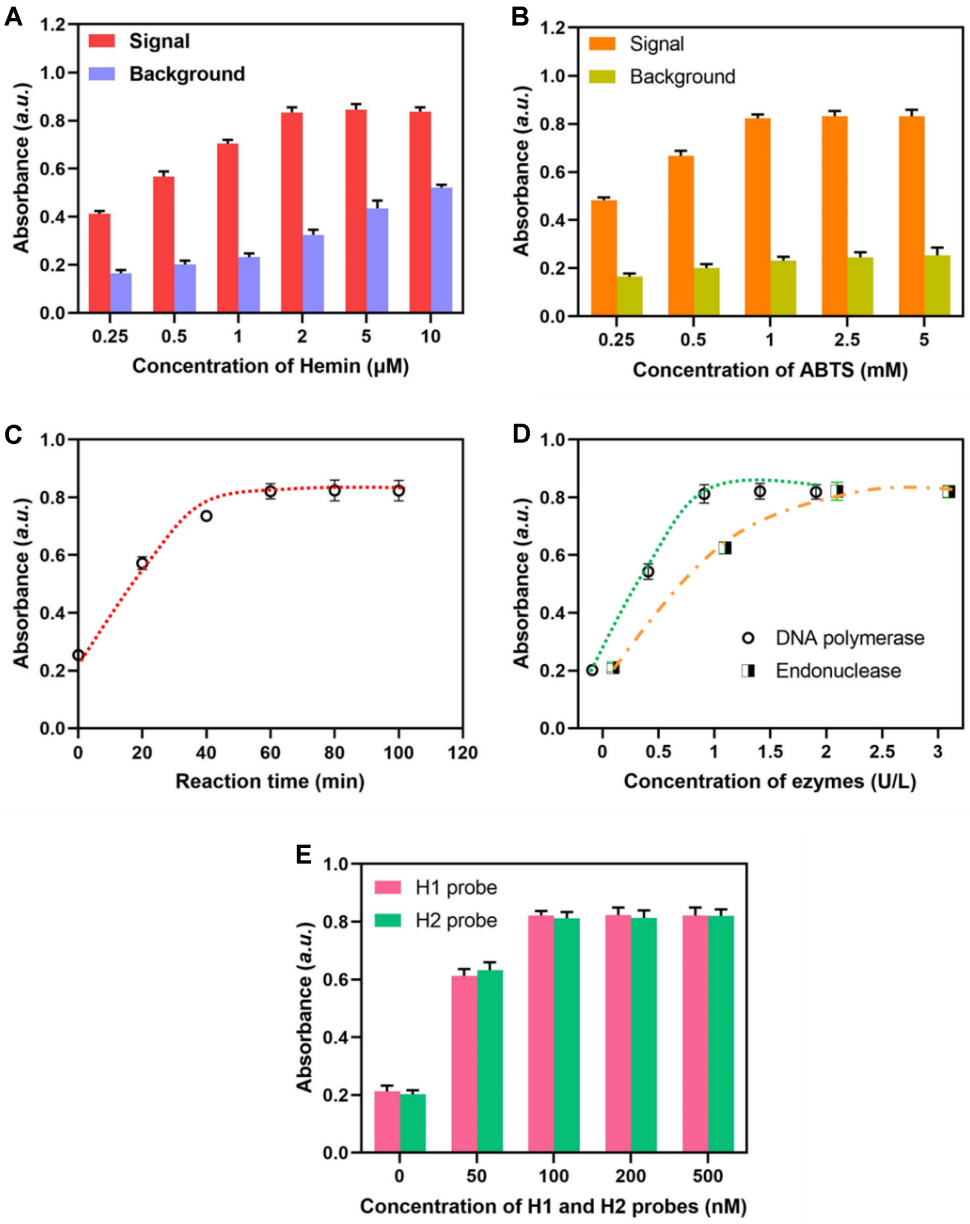
Optimization of experimental parameters. Absorbance of the approach when detecting MRSA with different Hemin concentration (**A**) ABTS concentration (**B**) reaction time (**C**) enzymes concentration (**D**) and probes concentration (**E**).

The presence of the unbound hemin molecule may initiate a minor oxidation reaction of ABTS^2−^, leading to the generation of a background signal. Consequently, the optimal concentrations of hemin and ABTS in the color reaction were initially evaluated to enhance the signal-to-background ratio. As depicted in Fig. 3A, the A415 exhibited a steady increase in the presence of MRSA, as the hemin concentration was raised, eventually reaching an equilibrium at 2 μM. In the absence of MRSA, the background absorbance exhibited a consistent upward trend as the concentration of hemin was augmented. Upon the addition of additional hemin, there was a rapid drop in the signal/background (S/B) ratio as the concentration of hemin increased from 1 to 10 μM. The best condition was determined to be a hemin concentration of 1 μM. The results depicted in Fig. 3B demonstrate a positive correlation between the ABTS concentration and both the signal and background levels. Specifically, as the ABTS concentration increases, there is a progressive rise in both the signal and background levels. This trend continues until the ABTS concentration reaches 1 mM, at which point the signal and background levels stabilize and remain constant. The reason for this is that the ABTS molecule functions solely as a substrate for the oxidation process. The presence of extra ABTS does not enhance the reaction rate of the hemin/ABTS/H_2_O_2_ system when compared to the system containing an equivalent amount of hemin/G-quadruplex enzymatic catalyst. Based on the equivalent signal-to-background ratio, it is shown that the signal obtained at an ABTS concentration of 2.5 mM is marginally greater compared to that at 1 mM. Consequently, an ABTS concentration of 2.5 mM was determined to be the ideal condition. Subsequently, an investigation was conducted to examine the impact of reaction duration, concentration of DNA polymerase, endonuclease, H1 probe, and H2 probe on the performance of the method. The study revealed that the A415 signal exhibited a rapid growth as the reaction time increased, ultimately reaching a plateau after approximately 60 min (see Fig. 3C). Fig. 3D and 3E illustrate the relationships between the final absorbance signal of the experiment and the amounts of DNA polymerase, endonuclease, H1 probe, and H2 probe, respectively. Therefore, the subsequent tests utilized an ideal concentration of 1 U/L of DNA polymerase, 2 U/L of endonuclease, and 100 nM concentrations of both the H1 and H2 probes.

### Sensitivity and Selectivity of MRSA Detection

**Fig. 4:**
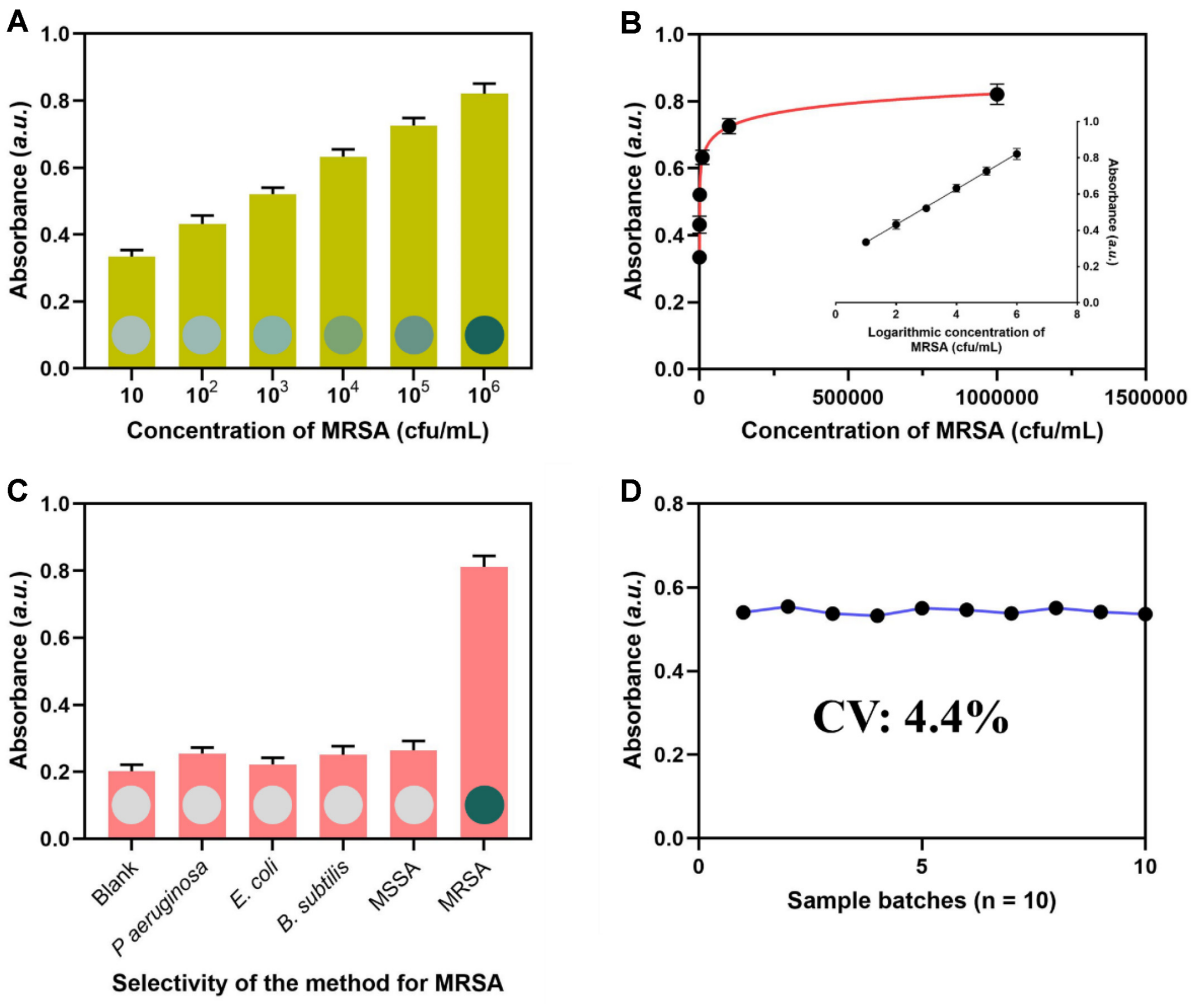
Detection performance of the proposed approach. (**A**) absorbance of the approach when detecting different concentrations of MRSA. (**B**) correlation between the calculated absorbance and the concentration of MRSA. (**C**) Absorbance of the approach when detecting MRSA and interfering bacteria. (**D**) Absorbance of the approach when detecting 10 sample batches.

The as-proposed procedure was utilized to validate the detection of MRSA spiked solutions at various concentrations, based on the optimal sensing circumstances. The discernible increase in blue coloration can be observed through visual examination, as depicted in Fig. 4A and 4B. The A415 value exhibited a rise within the concentration range of MRSA, ranging from 10 to 1.0 × 10^6^ CFU/ml. The A415 value demonstrated a strong linear relationship within the range of MRSA concentrations from 10 to 10^6^ CFU/ml, as indicated by a correlation coefficient of R^2^ = 0.996. The estimated detection limit was determined to be 3 CFU/ml, which was significantly lower compared to the detection limits observed in several colorimetric assays as stated in Table S1. In contrast to previously described techniques that integrate PBP2a aptamer and DNA amplifications to achieve comparable sensitivities, the cascade signal amplification strategy employed in this study significantly streamlines the manual procedures. There was no need for frequent washing of the MBs, addition of various reagents, or changing of the buffers. The signal amplification process may be fully executed within 60 min, indicating significant promise for on-site detection in the field of environmental monitoring.

Many non-target bacteria, such as *P aeruginosa*, *E. coli*, *B. subtilis*, and MSSA, were tested under the same conditions to further attest to the specificity of the current colorimetric MRSA detection. The absence of microorganisms in a control sample was carefully documented. Compared to the target MRSA (Fig. 4C), the absorbance of the samples containing other bacteria and the control is substantially lower. This demonstrates the high specificity. In addition, the MICs of the MRSA were determined by the E-test method, and the MRSA strains with different MICs were analyzed by the method. In detail, the MRSA strains were divided into three groups according to the different MICs of methicillin determined by the E-test method, including the high level (H), middle level group (M), and low level group (L). The amounts of the MRSA strains used in this experiment were all 5×10^3^ CFU/ml. The established approach was then utilized to detect the MRSA in these groups. The result in Fig. S3 showed that the proposed method could effectively distinguish the three groups. In order to assess the repeatability of the system, 10 batches were tested using the same concentration of MRSA (1.0 × 10^3^ CFU/ml). The coefficients of variation (CV) were determined to be 4.4% (Fig. 4D), suggesting that the reproducibility of the detection system was deemed satisfactory.

### Real Sample Measurements

**Fig. 5:**
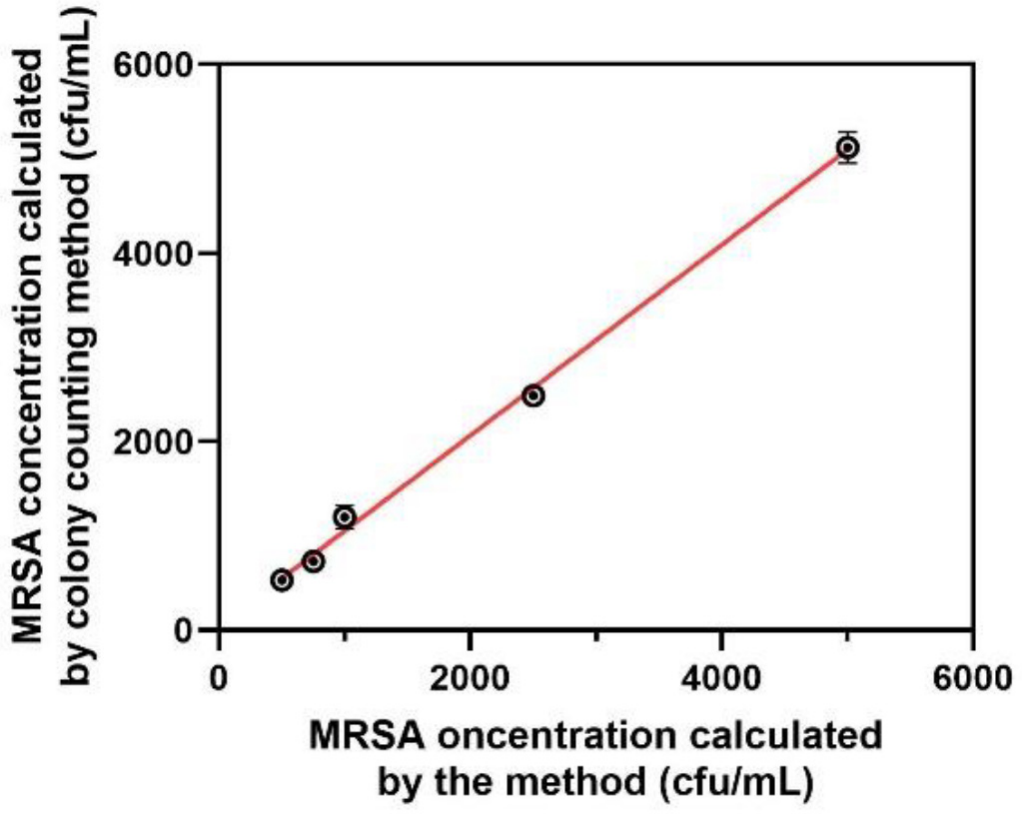
Clinical application of the proposed approach. MRSA concentration calculated by colony counting method (CFU/ml) and the proposed method.

In order to assess the reliability of this assay for practical sample applications, we conducted an experiment to detect various concentrations of MRSA (ranged from 100 to 1,000 CFU/ml) from PJI samples (specifically blood samples) using the proposed method and traditional colony counting method. As depicted in Fig. 5, the amounts of MRSA calculated by the proposed approach is closely aligned with the colony counting approach. Given that the majority of colorimetric assays utilized for detecting MRSA have been either semi-quantitative or quantitative in nature, the sensitivity and precision exhibited by our approach are deemed satisfactory for an on-site analysis. The results collectively demonstrate that the self-priming cascade signal amplification technique possesses favorable attributes such as selectivity, accuracy, consistency within a laboratory setting, and substantial resistance to interference from the matrix in actual samples. The results demonstrate significant promise for the implementation of on-site MRSA identification in complex samples, with potential for practical applications.

## Conclusion

In brief, we developed a rapid and highly sensitive colorimetric technique for the visual analysis of methicillin-resistance of *S. aureus* in clinical samples (blood samples obtained from patients with prosthetic joint infections). The utilization of an allosteric probe for precise target recognition, in conjunction with a cascade signal amplification, was implemented. The PBP2a aptamer in the allosteric probe successfully identified the target MRSA, leading to the initiation of self-primer elongation based-cascade signal amplification. The isothermal and self-sustaining production of peroxidase-like hemin/G-quadruplex effectively facilitates the oxidation of ABTS^2-^and produces a distinct blue hue, enabling the visual identification of MRSA at low concentrations. In contrast to the colorimetric approach that has been published, which exhibits limited sensitivity, the utilization of robust isothermal signal amplification in this sensor enables it to possess a wide dynamic range spanning from 1 to 1.0 × 10^6^ CFU/ml. Additionally, the sensor achieves a low detection limit of 3 CFU/ml. The implementation of cascade signal amplification resulted in a significant reduction in manual operations. In addition, the sensor has notable selectivity and reproducibility, demonstrating excellent performance when tested with authentic samples. Given the aforementioned benefits, it is evident that this colorimetric assay exhibits considerable promise for incorporation into a sensing kit, hence offering a more viable option for the swift and on-site analysis of methicillin-resistance in patients with prosthetic joint infections. In addition to the MRSA detection, the method could be applied for the methicillin-resistance analysis of non-aureus staphylococci, thus guiding the antibiotic selection in treatment and nursing of SSTIs. However, the method was designed to quantify the MRSA cells other than quantify the methicillin MICs (minimum inhibitory concentration) of MRSA. In the future, we will focus on developing novel methods that could specifically determine the methicillin MICs.

## Supplemental Materials

Supplementary data for this paper are available on-line only at http://jmb.or.kr.



## Figures and Tables

**Table 1:** Sequences of the oligonucleotides used in this research.

Title	Sequences (5’ to 3’)
H1 probe	CAC CCC ACC TCG CTC CCG TGA CAC TAA TGC TAT TTT TT T TTA TTT CTT TAT CCT CAG CTT AGT GTC ACTA AGC
H2 probe	TTT ATT TCT TTA TCC TCA GCTC CCT ATC CCT ATC CCT ATC CCT ACC TCA GCA AAG AAA TTT GCT GA
