# Supplementary material for: Allosteric Probe-Based Colorimetric Assay for Direct Identification and Sensitive Analysis of Methicillin Resistance of Staphylococcus aureus
Source: J Microbiol Biotechnol. 2024 Feb 19;34(3):681–8. doi: 10.4014/jmb.2312.12042 (PMC11016754; doi:10.4014/jmb.2312.12042)
Supplement: Supplementary file 1 [file jmb-34-3-681-supple.pdf]

J. Microbiol. Biotechnol.
https://doi.org/10.4014/jmb.2312.12042

Supplementary Table and Figures

Table S1. A brief comparison of the approach with former ones.

Title	Target	Mechanism	LOD	MICs analysis	Equipment	Extraction	Ref
The method	PBP2a	Chain displacement	3 cfu/mL	No	Naked eyes	-	
Dual-functional aptamer	PBP2a	CRISPR-Cas12a	100 cfu/mL	No	Fluorospectro photometer	-	[1]
Dual-Toehold-Probe-Mediated method	*mecA*	Exo-III assisted signal amplification	4.36 fM	No	Fluorospectro photometer	+	[2]
Ag^{+} Aptamer-Based Color Reaction	PBP2a	Polymerase-assisted target recycle	54 cfu/mL	No	Naked eyes	-	[3]

PBP2a, penicillin-binding protein 2a; Exo-III, Exonuclease-III; MICs, minimum inhibitory concentration; -, extraction free; +, need extraction.

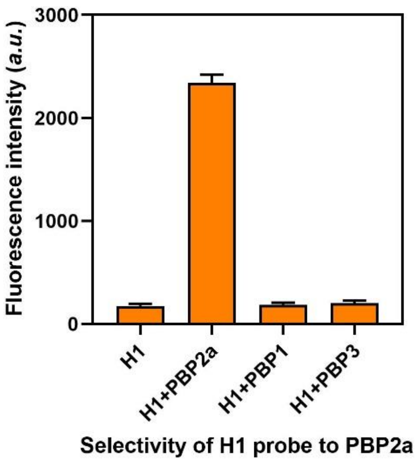

Fig. S1. Fluorescence intensities of the FAM labeled H1 probe when PBP2a, PBP1, PBP3 exited or not.

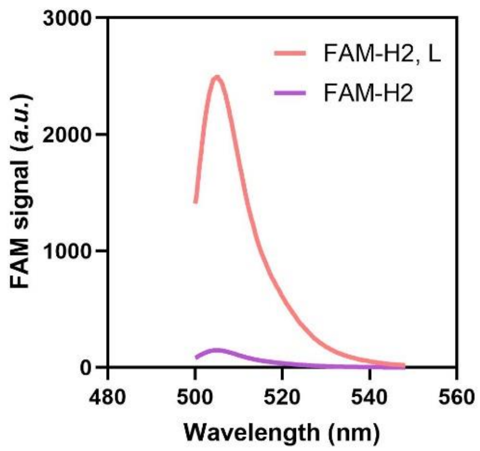

Figure S2. Fluorescence intensities of the FAM labeled H2 probe before and after assembly to hairpin structure.

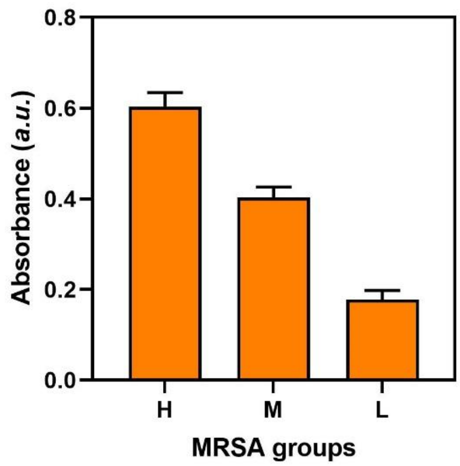

Figure S3. Absorbance of the approach when detecting MRSA with different methicillin resistance capability. The definition of low-medium-high level groups were high: more than 4 μg/ mL; middle: 1.5~3 μg/ mL; low: lower than 1 μg/ mL.

References

1. Xu L, Dai Q, Shi Z, Liu X, Gao L, Wang Z*, et al.* 2020. Accurate MRSA identification through dual-functional aptamer and CRISPR-Cas12a assisted rolling circle amplification. *J. Microbiol. Methods.* **173:** 105917.
2. Su J, Zheng W. 2023. Dual-Toehold-Probe-Mediated Exonuclease-III-Assisted Signal Recycles Integrated with CHA for Detection of mecA Gene Using a Personal Glucose Meter in Skin and Soft Tissue Infection. *J. Microbiol. Biotechnol.* **33:** 1692-1697.
3. Cao H, Zhang G, Ma H, Xue Z, Huo R, Wang K*, et al.* 2023. Sensitive and Extraction-Free Detection of Methicillin-Resistant Staphylococcus aureus Through Ag(+) Aptamer-Based Color Reaction. *J. Microbiol. Biotechnol.* **34:** 1-6.
